# Antidiabetic Effects of Corni Fructus Extract on Blood Glucose and Insulin Resistance in db/db Mice

**DOI:** 10.5487/TR.2009.25.2.093

**Published:** 2009-06-01

**Authors:** Hye Jeong Kim, Kil-Soo Kim, Tae-Jong Lee, Young Chul Kim

**Affiliations:** 15 Department of Public Health, College of Natural Sciences, Keimyung University, 2800 Dalgubeoldaero, Dalseo-gu, Daegu, 704-701 Korea; 25 Division of Human Blood Safety Surveillance, Korea Centers for Disease Control & Prevention, Seoul, 122-701 Korea; 35 College of Veterinary Medicine, Kyungpook National University, Daegu, 702-701 Korea; 45 Dept. of Pathology, Daegu Fatima Hospital, Daegu, 701-600 Korea

**Keywords:** Corni Fructus, Blood glucose, Insulin resistance, GLUT 4, db/db Mice

## Abstract

This study investigated the effect of Corni Fructus (*Cornus officinalis* Sieb, et Zucc.) extract on blood glucose and insulin resistance in db/db mice. Seven weeks old male mice were divided into normal control group (NC), diabetic control group (DC) and Corni Fructus treated diabetic group (DCF). Over an 8-week experimental period, Corni Fructus extract was administered orally at 500 mg/kg BW/day. Corni Fructus inhibited increase in blood glucose level during the OGTT (oral glucose tolerance test). At 8 weeks after beginning of the experiment, blood glucose level in the DCF group was significantly lower (*p*<0.01) than the DC group. Final fasting serum glucose and triglyceride in the DCF group were significantly lower (*p*<0.05) than the DC group by 32% and 41% respectively. Serum insulin did not differ among the NC, DC and DCF groups. The mRNA expression of adiponectin, GLUT 4 and PPAR-γ in adipose tissue in the DC group were significantly lower than the NC group and they were higher in the DCF group than the DC group by 76%, 130% (*p*<0.05) and 43%, respectively. In conclusion, these results indicated that Corni Fructus would have antidiabetic effects via improving insulin resistance in favor of higher glucose utilization and reducing blood glucose level in db/db mice.

## INTRODUCTION

Diabetes mellitus is the complex metabolic disease caused by an absolute or relative lack of insulin and resulting in deleterious effects on both the macrovascular and microvascular systems (Zimmet *et al.*, 2001). Insulin deficiency due to autoimmunity mediated depletion of pancreatic β-cells is considered as the etiology of type I diabetes mellitus (insulin dependant diabetes mellitus; IDDM). In contrast, both impaired insulin secretion and insulin resistance are two main characteristics for type II diabetes mellitus (non-insulin dependant diabetes mellitus; NIDDM) (Pickup and Williams, 2003). Diabetes mellitus is a chronic disease that cannot be completely cured and may develop various complications if not properly treated.

Sulfonylurea series drugs as hypoglycemic agent used widely for treatment of type II diabetes mellitus at present, if administered *in vivo* on a long-term basis, they may involve the exhaustion of β-cell as well as potential risks of hypoglycemia. Besides, metformin (beguanide series drug) may involve lactic acidosis as an adverse reaction (Bailey, 1999). Therefore, it is an increasing demand for natural products and traditional herbal medicines which have antidiabetic activities (Kim *et al.*, 2008).

Fructus of *Cornus officinalis* Sieb. et Zucc. (Corni Fructus) has been used as Korean traditional medicine. It represents one of the seven-component herbs in Yukmi-jihang-tang that has been used for the treatment of diabetes mellitus or diabetic complications in Korean traditional medicine (Jin *et al.*, 2006). Recently, it has been reported that Corni Fructus has beneficial effect on advanced glycation end product-mediated renal injury in STZ-treated diabetic rats (Yamabe *et al.*, 2007). However, beneficial effects of Corni Fructus on the hyperglycemia and insulin resistance in db/db mice have not yet to be explored.

Since it was already reported that insulin resistance is a common factor inducing hypertension, diabetes and obesity (Reaven, 1993), the treatment of insulin resistance has been the greatest challenge in treatment of type II diabetes. Thiazolidinediones (rosiglitazone, pioglitazone) series drugs used commonly for type II diabetes have been lately known to improve insulin resistance in animal and human (Saltiel and Olefsky, 1996). PPAR-$\gamma$ is a molecular target of thiazolidinediones and is also one of nuclear receptor superfamilies (Mangelsdorf *et al.*, 1995). Some studies reported that rosiglitazone activates PPAR-$\gamma$ to enhance the glucose availability and also increases the concentration of adiponectin as a marker of insulin sensitivity (Yu *et al.*, 2002). Insulin-induced blood glucose lowering actions are represented by glucose uptake in skeletal muscle and fat, which were resulted from the mobilization of GLUT4 into the membrane. Dysfunction of this mechanism is one of the major causes of provoking insulin resistance (Hansen *et al.*, 1995).

This study investigated the effects of Corni Fructus extract on the blood glucose and insulin resistance in db/db mice, and intended to measure the expression of GLUT4, PPAR-$\gamma$ and adiponectin as the markers of insulin resistance.

## MATERIALS AND METHODS

***Corni Fructus water extraction.*** The fruits of *Cornus officinalis* were collected from Gunwi, Gyeongbuk, Korea and authenticated by a Doctor of Oriental Medicine in Department of Oriental Medicine, College of Oriental Medicine, Sangji University (Gangwon-Do, Korea). Six-hundred gram of Corni Fructus (fruits of *Comus officinalis*) with 6 l distilled water was boiled for 2 hours in a heating extractor (COSMOS-660, Kyungseo Machine Co., Korea) and concentrated to 3 l. Thereafter, the aqueous extract was distributed into pouches containing daily volume each and stored at 4°C until to use. The calculated yield of the water extract was 25% (w/w) by a lyophilization method.

***Animals and experimental design.*** Seven weeks male C57BL/KsJ-db/db mice and C57BL/6 mice (non-diabetic mice) were purchased from the Central Lab. Animal Inc. (Seoul, Korea). Animals were housed in plastic cages at 22 ± 1°C, with a relative humidity of 50 ± 5%, an alternating 12 h light/dark cycle, and were allowed to have access to their respective diets *ad libitum.* The animals were allowed to acclimatize to the laboratory environment for 7 days and then were randomly assigned to one of three groups (seven animals each), for the 8-week experiment. The experimental groups were as follows:

Group I, non-diabetic control C57BL/6 mice (NC)

Group II, diabetic control db/db mice (DC)

Group III, diabetic db/db mice fed Corni Fructus extract (DCF).

For repeated oral administration, mice were treated once daily, 6 days a week, for 8 weeks. Group I and II received distilled water, Group III received the water extract at 10 ml/kg BW/day (500mg extracted powder of Corni Fructus/kg BW/day). The dosage of Corni Fructus in this study was adopted based on traditional prescriptions in oriental medicine. Body weight and blood glucose level were monitored weekly between nine and ten o'clock in the morning. Daily food and water consumption were monitored weekly and were determined by subtracting left-over amount from the total amount provided. Blood was withdrawn from the tail vein and used for plasma glucose determination using the glucose oxidase method with a Gluco card II™ (ARKRAY, Japan). Removed adipose tissues from intestinal tracts were frozen in liquid nitrogen and stored at -80°C for genetic analysis, and removed pancreas were fixed in 10% neutralized buffered formalin solution for immunohistochemical analysis.

***Oral glucose tolerance test.*** Oral glucose tolerance test was performed in all animals in each group. After 7 weeks treatment of Corni Fructus, a set of blood samples, following 6 h of fasting were taken from all groups. Blood samples were collected from the tail vein at 0, 30, 60, 90 and 120 min intervals after the oral administration of 2 g glucose/kg BW for the determination of glucose level.

***Blood analysis.*** At 8 weeks after beginning of the experiment, 4 h fasting blood samples were collected from the posterior vena cava under ether anesthesia. The serum glucose and triglyceride were measured using a commercial available assay kit (EIKEN, Japan) with a Hitachi-7600 Analysis System (Hitachi, Japan) by the method of Brandstrup *et al.* (1957) and McGrown *et al.* (1983). Serum insulin was measured using a mouse insulin ELISA kit (Mercodia, USA) with a microplate reader (Molecular devices, USA).

***Isolation of total RNA and cDNA synthesis.*** Total RNA was isolated from adipose tissue of mice using the High Pure RNA Isolation Kit (Roche Applied Science, Penzberg, Germany) following the manufacturer's protocol. The quantity and quality of the isolated total RNA were determined by the UV/Vis spectrophotometer

(Mecasys Co., Korea). Only samples with $2.0>OD_{260/280}>1.8$ were processed. cDNA was synthesized from 2 μg of total RNA, using AccuPower CycleScript RT PreMix Kit (Bioneer, Korea) in a final volume of 20 μl.

***Real-time quantitative RT-PCR***. Quantitative real-time RT-PCR was used to quantify the amounts of each gene mRNA. cDNA was diluted 1 : 10 with autoclaved deionized water, and 2 μl of the diluted cDNA was added to 10 μl of iQ™ SYBR Green Supermix (BioRad Laboratories, Inc, USA) and 7 pmol/l specific primer. This reaction mixture was filled up to a final volume of 20 μl with water. PCR was carried out in a real-time PCR cycler (iCycler IQ System, BioRad, USA). The program was optimized and performed finally as denaturation at 95°C for 5 min followed by 40 cycles of amplification (95°C for 20 s, annealing Tm for 20 s, 72°C for 30 s). After completion of the PCR, the melting curve of the product was measured by temperature gradient from 60 to 95°C at 0.5°C per second with continuous fluorescence monitoring to produce a melting profile of the primers. The relative abundance of mRNA was standardized with β-actin mRNA as the invariant control. The amplification curves for Ct (threshold cycle) determination, and melting curves for temperature estimations at the peak of the curves, were analyzed using the Gene Expression Analysis for iCycler iQ Real-Time PCR Detection System from BioRad. Primer specific for the genes studied are listed in Table 2.

***Immunohistochemistry.*** Immunohistochemical analysis of insulin was performed on formalin-fixed paraffin-embedded pancreatic tissue using the BenchMark XT automated immunostainer (Ventana Medical Systems, USA). Briefly, tissue sections were incubated for 32 min with rabbit polyclonal anti-mouse insulin antibodies (Novus Biologicals, USA). Sections were incubated for 8 min with Biotinylated goat anti-rabbit IgG (Ventana Medical Systems, USA), followed by incubation for 8 min with avidin-HRP and were then developed with DAB. Sections were finally counterstained in Mayer's hematoxylin and mounted. Non-immune rabbit IgG was used as the negative control.

***Statistical analysis.*** Values were given as means ± SD of 7 mice in each group. The data were analyzed by Student's t-test using SPSS-12.0. The limit of statistical significance was set at $p<0.05$.

## RESULTS

***Water and food intakes, body weight gain and food efficiency ratio.*** Water intake in DC and DCF groups were significantly higher ($p<0.001$) than the NC group by 222% and 213%, respectively and it did not differ between the two diabetic groups. Food intake in DC and DCF groups were significantly higher ($p<0.001$) than the NC group by 90% and 73%, respectively and that of the DCF group was significantly lower ($p<0.01$) than the DC group. Body weight gain in DC and DCF groups were higher than the NC group and it was higher in the DCF group than the DC group by 33%. Food efficiency ratio in the DC group was lower than the NC group and it was higher in the DCF group than the DC group by 51% (Table 1).

***Oral Glucose Tolerance Test (OGTT).*** The effect of Corni Fructus on changes in blood glucose levels during OGTT in db/db mice is shown in Fig. 1. The levels of blood glucose obtained 90 and 120 min after glucose intake were significantly lower ($p<0.05$) in the DCF group than the DC group.

**Table 1.** Water and food intakes, body weight gain, food efficiency ratio, levels of serum glucose, insulin and triglyceride of C57BL/6 and db/db mice fed the Corni Fructus extract for 8 weeks

| Items | NC | Diabetic groups[a] | |
|---|---|---|---|
| | | DC | DCF |
| Water intake (ml/day) | 14.20 ± 1.41 | 45.72 ± 3.62[###] | 44.46±3.20[###] |
| Food intake (g/day) | 3.88 ± 0.13 | 7.37 ± 0.17[###] | 6.70±0.52[###] ** |
| Body weight gain (g/day) | 0.10 ± 0.01 | 0.15 ± 0.07 | 0.20±0.09[#] |
| Food efficiency ratio[b] (%) | 2.51 ± 0.33 | 2.11 ± 0.92 | 3.19±0.84 |
| Serum glucose (mg/dl) | 175.58 ± 11.90 | 776.53 ± 190.63[###] | 531.75±100.43[###] * |
| Serum insulin (pmol/) | 488.47 ± 115.76 | 590.58 ± 110.48 | 696.97±240.46 |
| Serum triglyceride (mg/dl) | 63.36 ± 13.70 | 317.16 ± 78.65[###] | 186.95±50.03[###] * |

[a]NC: Non-diabetic control group; DC: Untreated diabetic group; DCF: Diabetic group fed Corni Fructus extract
[b]Food efficiency ratio = (body weight gain/food intake) × 100.
Values are means ± SD of 7 mice.
The value with a sharp-note is significantly different from NC group by t-test (#; $p<0.05$, ###; $p<0.001$).
The value with an asterisk is significantly different from DC group by t-test (*; $p<0.05$, **; $p<0.01$).

**Table 2.** Nucleotide sequences for quantitative real-time PCR

| Gene name | | Primer sequences | Tm[a] (°C) | Accession No. |
|---|---|---|---|---|
| β-actin[c] | F[b] (53)<br>R (53) | CCCAGGCATTGCTGACAGG<br>TGGAAGGTGGACAGTGAGGC | 55~59 | X 03672 |
| GLUT4[d] | F (53)<br>R (53) | TGGTGTGGTCAATACGGTCTTC<br>GTTCCAGCAGCAGCAGAGC | 55 | NM 009204 |
| PPAR-γ[e] | F (53)<br>R (53) | CAGGCTTGCTGAACGTGAAG<br>GGAGCACCTTGGCGAACA | 55 | NM 011146 |
| Adiponectin | F (53)<br>R (53) | ACAAGGCCGTTCTCTTCACC<br>CCCCATCCCCATACACCTG | 55 | NM 009605 |

[a]Optimal annealing temperature
[b]F: forward, R: reverse
[c]House keeping gene
[d]Glucose transporter-4
[e]Peroxisome proliferator-activated receptor γ

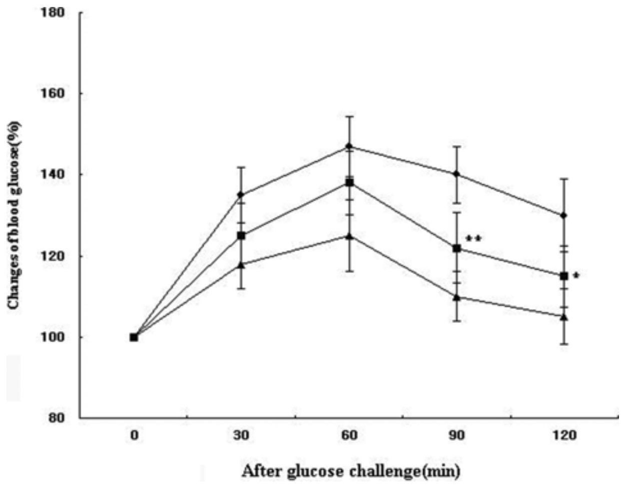

**Fig. 1.** Changes in blood glucose levels during the OGTT of C57BL/6 and db/db mice fed the Corni Fructus extract for 7 weeks. All mice fasted for 6h before OGTT (oral glucose tolerance test). Blood was taken from the tail vein at 0, 30, 60, 90 and 120 min after the oral administration of 2 g glucose/kg BW. Glucose concentration was determined by the glucose oxidase method. Values are means ± SD of 7 mice. The value with an asterisk is significantly different from DC group by t-test (*; $p$<0.05, **; $p$<0.01). ▲; NC, ●; DC, ■; DCF.

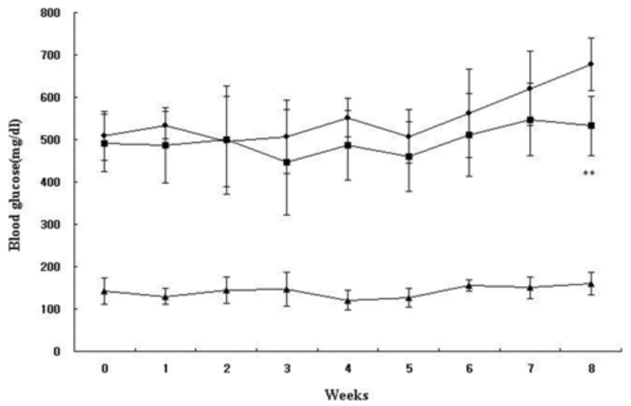

**Fig. 2.** Changes in blood glucose levels of C57BL/6 and db/db mice fed the Corni Fructus extract for 8 weeks. Values are means ± SD of 7 mice. The value with an asterisk is significantly different from DC group by t-test (**; $p$<0.01). ▲; NC, ●; DC, ■; DCF.

***Fasting serum glucose, insulin and triglyceride levels.*** A significant elevation ($p$<0.001) in final fasting serum glucose level was observed in diabetic groups compared with the NC group and it was significantly lower ($p$<0.05) in the DCF group than the DC group by 32%. Serum insulin levels did not significantly differ among the NC, DC and DCF groups. Serum triglyceride level in the DC group was significantly higher ($p$<0.001) than the NC group. However, it was significantly lowered ($p$<0.05) in the DCF group compared with the DC group by 41% (Table 1).

***Blood glucose levels.*** Blood glucose level in the diabetic groups were significantly higher ($p$<0.001) than the NC group throughout the experimental period. At 8 weeks after beginning of the experiment, it was significantly lower ($p$<0.01) in the DCF group than the DC group (533 ± 69.9 vs. 678 ± 61.8) (Fig. 2).

***Adiponectin, GLUT 4 and PPAR-γ expression in adipose tissue by real-time quantitative RT-PCR.*** The mRNA expression of adiponectin in the DC group was significantly lower ($p$<0.001) than the NC group (4.65 ± 0.74), and it was slightly higher in the DCF group than the DC group (0.60 ± 0.39 vs. 0.3 ± 0.16). The mRNA expression of GLUT 4 in the DC group was significantly lower ($p$<0.01) than the NC group (3.26 ± 0.92), and it was significantly higher ($p$<0.05) in the DCF group than the DC group (1.61 ± 0.57 vs. 0.70 ± 0.56). The mRNA expression of PPAR-γ in the DC

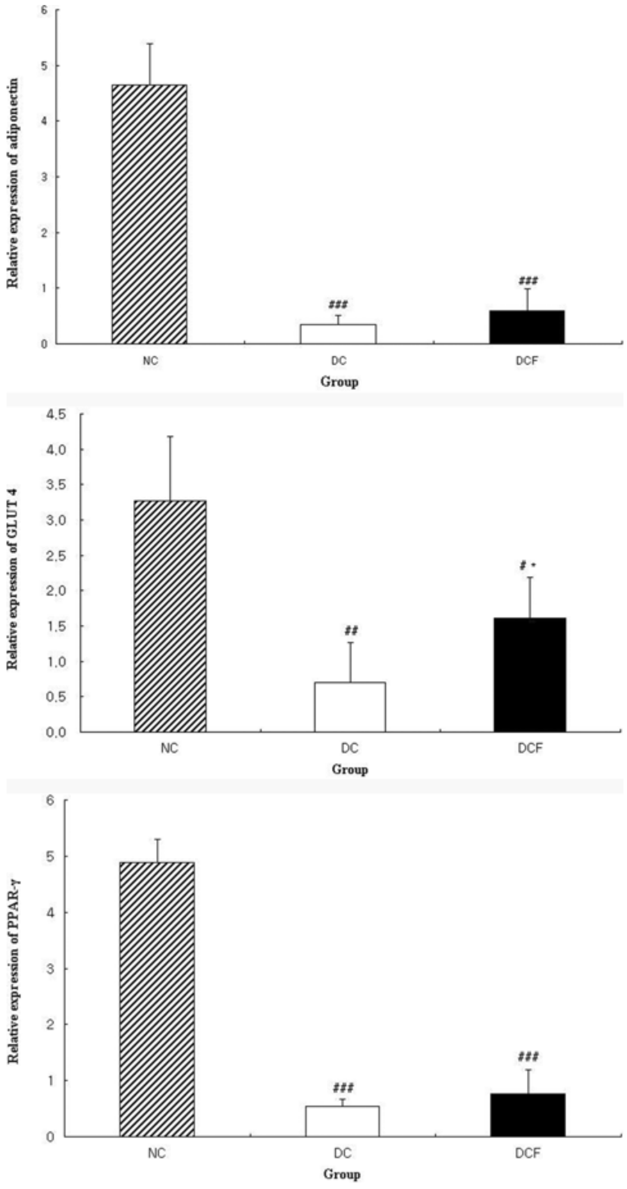

**Fig. 3.** Adiponectin, GLUT 4 and PPAR-γ expression in adipose tissue of C57BL/6 and db/db mice fed the Corni Fructus extract for 8 weeks. Values are means ± SD of 5 mice, representing relative ratios to the housekeeping gene (β-actin) by real-time RT-PCR. The value with a sharp-note is significantly different from NC group by t-test (#; $p$<0.05, ##; $p$<0.01, ###; $p$<0.001). The value with an asterisk is significantly different from DC group by t-test (*; $p$<0.05). NC: Non-diabetic control group; DC: Untreated diabetic group; DCF: Diabetic group fed Corni Fructus extract.

group was significantly lower ($p$<0.001) than the NC group (4.88 ± 0.42), and it was slightly higher in the DCF group than the DC group (0.77 ± 0.43 vs. 0.54 ± 0.13) (Fig. 3).

***Immunohistochemical analysis of insulin.*** Overall, immunohistochemical staining for insulin did not differ among the NC and diabetic groups. We could not observe a discernible difference in islet structure (Fig. 4).

## DISCUSSION

Despite the latest great academic advancement of life science, the incidence rate of diabetes mellitus and the mortality have been gradually increasing due to diabetic complications. There are many academic interests focused on possible ways to prevent and to remedy diabetes mellitus, but contemporary medicine has still failed to develop any fundamental therapeutics. There are many recent studies focused positively on therapeutics of diabetes mellitus using traditional herbal medicines or natural functional foods.

This study adopted oral administration of water extract of Corni Fructus, a kind of Korean traditional medicinal herb, into db/db mice as type II diabetic model to find out beneficial effects on improving hyperglycemia and insulin resistance. Animals were divided into 3 groups, i.e. normal control group (NC), diabetic control group (DC) and Corni Fructus treated diabetic group (DCF). NC group consisted of 7 male C57BL/6 mice, while DC and DCF groups consisted of respective 7 male db/db mice. Corni Fructus extract was orally administered at 500 mg/kg BW/day for 8 weeks.

According to the results in this study, it was found that diabetic groups showed remarkably higher water and food intakes than NC group, but there was no finding on any variation of water intake due to administration of Corni Fructus extract. DCF group had significantly lower dietary uptake than DC group, but DCF group showed higher body weight gain and food efficiency ratio than DC group. Thus, it was found that the administration of Corni Fructus extract has positive effects on improving degenerative changes of in vivo metabolism due to diabetes.

db/db mice show characteristics such as insulin resistance, hyperglycemia and obesity. According to the results of 8-week observation about blood glucose level, DC group began to show steep increase in blood glucose level from 6 weeks after beginning of the experiment. At 8 weeks treatment of Corni Fructus, DCF group showed significantly lower blood and serum glucose levels than DC group. Corni Fructus inhibited increase in the blood glucose level during the OGTT in db/db mice. Thus, it is found that Corni Fructus has positive effects on reducing blood glucose level and improving glucose tolerance.

Insulin is combined with insulin receptor in muscle

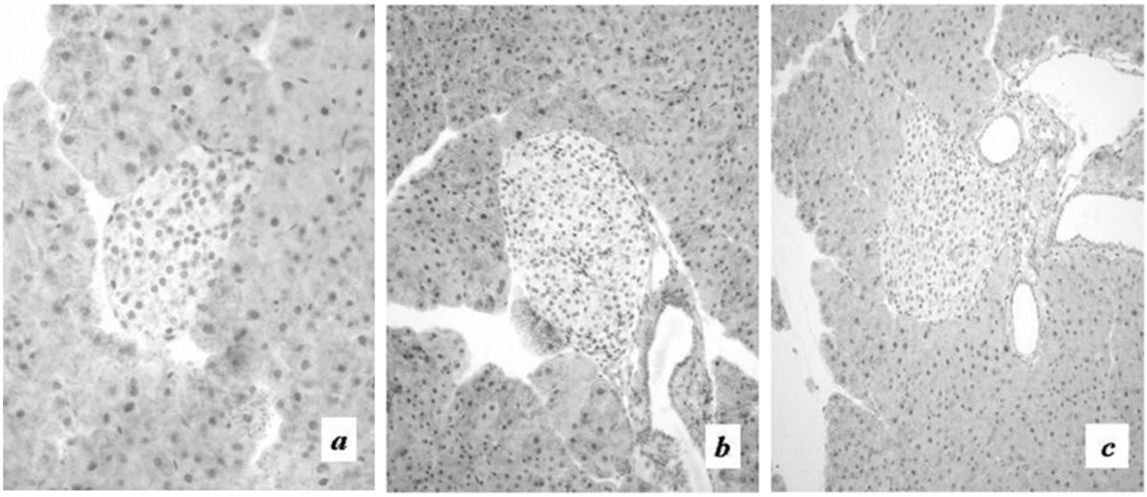

**Fig. 4.** Immunohistochemical staining for insulin in pancreas of C57BL/6 and db/db mice fed the Corni Fructus extract for 8 weeks. 200 ×. (a) Non-diabetic control group (NC), (b) Untreated diabetic group (DC), (c) Diabetic group fed Corni Fructus extract (DCF).

and fat cell, then the translocation of GLUT4 as a glucose transporter is induced, which in turn results in enhancement of glucose uptake into cells (Kanzaki *et al.*, 2004). Chomczynski and Sacchi (Chomczynski and Sacchi, 1987) reported that the decrease in glucose uptake is a major contributor to inducing hyperglycemia and insulin resistance owing to reduction in GLUT4 level. Garvey *et al.* (Garvey, 1989) reported the reduction in mRNA and protein content of GLUT4 in muscular tissues of db/db mice. Similarly, GLUT4 mRNA expression in the adipose tissues of db/db mice was remarkably reduced in this study, and GLUT4 mRNA level in DCF group was significantly higher than DC group.

Adiponectin, which is another hormone secreted from adipose tissue, works to increase susceptibility to insulin, contributes crucially to improve glucose metabolism and insulin resistance, and it is reduced in obesity or type II diabetes (Shojima *et al.*, 2002; Ryan *et al.*, 2003). It was found that the expression of adiponectin mRNA was remarkably reduced in adipose tissues of db/db mice compared with normal C57BL/6 mice in this study, and DCF group showed a little higher adiponectin mRNA level than DC group.

PPAR-γ is a receptor to control lipid homeostasis, lipocyte differentiation, insulin action, etc, and also associated with improving in metabolic syndrome such as insulin resistance, obesity and hyperlipidemia (Combs *et al.*, 2002). The expression of PPAR-γ mRNA in adipose tissues of db/db mice in this study was remarkably reduced, and DCF group showed a little higher mRNA level than DC group. Up-reguratory action of Corni Fructus on PPAR-γ mRNA expression was reconfirmed by significant reduction in serum triglyceride level of DCF group compared with DC group.

In this study, it was found that there was no significant difference in serum insulin levels among all 3 experimental groups. In the immunohistochemical staining for insulin, it was also found that there was no significant difference in both the morphologic changes in pancreas and the extent of insulin expression among these 3 groups. Corni Fructus had no significant influence on insulin secretion, however, DCF group showed a significant decrease in blood and serum glucose levels. Thus, it is estimated that Corni Fructus facilitates expression of PPAR-γ in adipose cells, which increases glucose uptake through the improvement of insulin resistance due to increase in expression of GLUT4 and adiponectin, and ultimately contribute to improving hyperglycemia.

In conclusion, these results indicated that Corni Fructus would have antidiabetic effects via improving insulin resistance in favor of higher glucose utilization and reducing blood glucose level in db/db mice.

## ACKNOWLEDGEMENTS

This work was supported by a grant of the Research Program of The Health Fellowship Foundation, Seoul, Korea.
